# Novel Thermotolerant Amylase from *Bacillus licheniformis* Strain LB04: Purification, Characterization and Agar-Agarose

**DOI:** 10.3390/microorganisms9091857

**Published:** 2021-09-01

**Authors:** Anaid Silva-Salinas, Melissa Rodríguez-Delgado, Jesús Gómez-Treviño, Ulrico López-Chuken, Clarita Olvera-Carranza, Edgar Allan Blanco-Gámez

**Affiliations:** 1 Centro de Investigación en Biotecnología y Nanotecnología (CIByN), Facultad de Ciencias Químicas, Universidad Autónoma de Nuevo León, Parquede Investigación e Innovación Tecnológica, Km. 10 Autopista al Aeropuerto Internacional Mariano Escobedo, Apodaca C.P. 66629, Nuevo León, Mexico; anaid.silva.salinas@uanl.edu.mx (A.S.-S.); melissa.rodriguezdl@uanl.edu.mx (M.R.-D.); ulrico.lopezch@uanl.mx (U.L.-C.); 2 Laboratorio de Biología Molecular, CELAES, Facultad de Ciencias Químicas, Universidad Autónoma de Nuevo León, San Nicolás de los Garza C.P. 66455, Nuevo León, Mexico; jesus.gomeztrv@uanl.edu.mx; 3 Departamento de Ingeniería Celular y Biocatálisis, Instituto de Biotecnología, Universidad Nacional Autónoma de México, Av. Universidad 2001, Chamilpa, Cuernavaca C.P. 62210, Morelos, Mexico; clarita@ibt.unam.mx

**Keywords:** thermostability, α-amylase, agar-agarose immobilization, *Bacillus licheniformis*, hot springs, ion exchange chromatography, free enzyme, immobilized α-amylase

## Abstract

This study analyzed the thermostability and effect of calcium ions on the enzymatic activity of α-amylase produced by *Bacillus licheniformis* strain LB04 isolated from Espinazo Hot springs in Nuevo Leon, Mexico. The enzyme was immobilized by entrapment on agar-agarose beads, with an entrapment yield of 19.9%. The identification of the bacteria was carried out using 16s rDNA sequencing. The enzyme was purified through ion exchange chromatography (IEX) in a DEAE-Sephadex column, revealing a protein with a molecular weight of ≈130 kDa. The enzyme was stable at pH 3.0 and heat stable up to 80 °C. However, the optimum conditions were reached at 65 °C and pH 3.0, with a specific activity of 1851.7 U mg^−1^ ± 1.3. The agar-agarose immobilized α-amylase had a hydrolytic activity nearly 25% higher when compared to the free enzyme. This study provides critical information for the understanding of the enzymatic profile of *B. licheniformis* strain LB04 and the potential application of the microorganisms at an industrial level, specifically in the food industry.

## 1. Introduction

Starch is a polysaccharide composed of several glucose units bound together by α-1,4-glycosidic linkages and α-1,6-glycosidic bonds. Through the bond branching, starch occurs as granules composed of two main polymers of glucose: amylose and amylopectin [1]. While amylose is a linear chain of α-D-glucose units joined together by α-1,4-glycosidic linkages, amylopectin is a polysaccharide built by α-1,4 and α-1,6-glycosidic bonds, the latter being responsible for the branching inside starch granules. The disruption of starch granules to obtain glucose is known as starch hydrolysis, essential for a wide range of industrial products [2].

Acidic hydrolysis of starch was widely replaced by enzymatic hydrolysis since it performs under less demanding conditions, in addition to minimizing extreme parameters reached during chemical and physical treatments, such as temperature, pressure, and prolonged periods of time [3].

The α-amylase is an extracellular enzyme produced by a diversity of organisms that cleave into the α-1,4-glycosidic linkages inside the starch granules, releasing glucose monomers and several oligosaccharides, such as maltose [4]. Due to its versatility, α-amylase was used in a wide range of industrial sub-sectors, such as the textile, pharmaceutical, brewing, detergent, and baking industry. This enzyme alone represents more than 50% of the global demand in the enzyme market. For instance, the worldwide α-amylase market for the baking industry valued at USD 278.23 million in 2018, was estimated to reach USD 352.78 million by 2026 [5,6]. The α-amylase improves bread properties by attenuating its damaged starch content and increasing carbon dioxide production during dough fermentation. By changing the mixture’s rheology, a higher level of elasticity is reached, thus increasing bread volume and firmness and reducing the crumb ratio [7]. This enzyme was proved to decrease starch retrogradation levels, improve crumb formation during longer storage periods, and lower water-loss rates [8]. As enzymes help meet consumer demands of better quality bakery products, the need for cost-effective, enhanced performance, thermostable and versatile α-amylase enzymes that adjust to specific processes is essential.

Enzymes produced by thermophilic organisms are commonly identified as thermostable due to their performance capacity under high temperatures without losing catalytic efficiency through temperature shifting. Microorganisms able to endure and adapt to unusual environments, for instance, where temperature repetitively drops and rises, where pH values are irregular or there is continuous exposure to high levels of salts, minerals or even pollutants, become targets for research focused on the production of enzymes better-suited for industrial applications [9,10,11,12]. Enzyme immobilization is a well-known technique where the physicochemical properties of a compound acting as a support allow interaction with the structure of an enzyme. This interaction culminates with the rearrangement of the enzyme’s original configuration, achieving better enzyme stability during catalytic reactions, and simplifying its recovery for future reuse [13,14,15]. Agar-agarose, an acid-resistant polymer with a prolific gelling ability, was promoted as a carrier agent for different enzyme’s entrapment due its inert nature and low-cost production. These were the main reasons for choosing it as the immobilization matrix for the current study. The entrapment technique is easier to perform against covalent binding, since it allows thermo- and acid-sensitive proteins to preserve or even improve their enzymatic activity without interacting with the active site. The enzyme is captured within the polymeric matrix which allows a controlled diffusion while reacting with the substrate. At the same time, the enzyme suffers a reconfiguration in its structure, resulting in changes through its activity performance under different conditions, such as temperature, pH and substrate concentration [16].

The industrial need for better performing enzymes under harsh conditions and limited timeframes encourages the development of more efficient microorganisms. However, such microorganisms might already inhabit ecosystems that parallel said conditions. That is why in the present study, *Bacillus licheniformis* strain LB04 was isolated from a hot spring (average water temperature of 55 ± 1.0 °C), in Espinazo, Nuevo Leon State in Mexico, to determine its capacity to produce a thermostable α-amylase enzyme. Different experiments were carried out to evaluate its ability to hydrolyze starch under high temperatures and acidic conditions, while subtracting calcium ions from the reaction, due to their nature as structure stabilizing agents. The enzyme immobilization by physical entrapment in agar-agarose beads was employed as the main technique to study its performance in the interest of improving the enzymatic activity for potential industrial applications, such as starch degradation, in which reducing costs and performance improvement is imperative.

## 2. Materials and Methods

### 2.1. Chemicals

Difco Nutrient broth and Difco Starch agar BD Bioscience, JT Baker sodium chloride, PQM magnesium sulfate heptahydrate, sodium hydroxide, potassium sodium tartrate, DEAE-Sepharose and JT Baker calcium chloride were purchased from CTR Scientific (Nuevo Leon, Mexico). The JT Baker potassium iodate, JT Baker agar, and iodine solution were bought from Productos Quimicos Monterrey, S.A. de C.V. FERMONT (Nuevo Leon, Mexico). JT Baker Soluble starch, Bacto yeast extract, casein peptone and BIO-RAD protein assay dye reagent concentrate and bovine serum albumin standard were acquired from Insumos Biomoleculares, S.A. de C.V. (CDMX, Mexico). The 3,5-dinitrosalicylic acid (DNS) was purchased from Thermo Fisher Scientific Inc (CDMX, Mexico).

### 2.2. Sample Collection

Water and sediment samples were collected from a hot spring located in Espinazo, Nuevo Leon, Mexico [17] using 50 mL sterilized falcon tubes and stored at −20 °C for further use.

### 2.3. Microbial Strains and Medium Composition

Samples were grown in 250 mL of nutrient broth medium (beef extract 10.0 g, casein peptone 10.0 g, NaCl 5.0 g dissolved in 1 L deionized water; pH 7.3) in Erlenmeyer flasks. The flasks were incubated for 24 h at 45 °C under shaking conditions. A sample of 1.0 mL of the grown culture was transferred to starch agar plates (soluble starch 10.0 g, casein peptone 3.0 g, agar 12.0 g, pH 6.0; made with 1 L deionized water) for isolation and identification of the microbial species.

### 2.4. Screening of Amylase Producing Bacteria and Growth Conditions

The colonies isolated from starch agar plates were tested to produce α-amylase enzymes using the Lugol staining assay [18]. Only the bacteria species showing the highest α-amylase production were identified and repeatedly grown on a starch culture medium (soluble starch 10.0 g, yeast extract 3.0 g, casein peptone 5.0 g, NaCl 3.0 g, MgSO_4_ • 7H_2_O 0.5 g, CaCl_2_ 3.0 g, pH 5.5; made with 1 L deionized water) at constant shaking at 150 rpm and 45 °C [19]. The cell-free supernatant used as the α-amylase enzyme source was collected by centrifugation at 14,000 rpm for 40 min at 4 °C and tested for amylase activity through the DNS method.

### 2.5. Morphological, Biochemical, and Physiological Characterization

Morphology of the isolated strain was determined using the Gram staining technique. The endospore and capsule staining technique was also employed. Bacterial morphology was observed with a binocular microscope CxL (LABOMED). The biochemical characterization of the resulting enzyme activity was determined through different substrate degradation and resulting bio-products.

### 2.6. Molecular Phylogenetic Analyses

The 16s rDNA sequence was analyzed through the BLASTN data to identify the isolated α-amylase-producing strain. A neighbor-joining phylogenetic tree was constructed using MEGA6 software.

### 2.7. Amylase Activity Assay

The starch hydrolytic activity of the isolated strain was estimated using the DNS assay method [20] as follows: the enzymatic activity was measured using 50 µL of enzyme solution with 50 µL of starch solution 1.0% and incubated for 60 min at 45 °C. A 50 μL sample of the reaction was mixed with 50 μL of DNS solution. The mixture was incubated in a boiling water bath for 5 min and immediately cooled down for 10 min in an ice bath. The absorbance of the cooled mixture was measured at 540 nm using UV-vis spectrophotometry, where one unit of amylase activity was defined as the amount of enzyme that releases 1 μg/mL of maltose per minute under the assay conditions. Total protein concentration was determined by Bradford protein assay [21].

### 2.8. Preparation of Crude Extract for Purification of α-Amylase

A fermented media (48 h, 150 rpm, 45 °C) was centrifuged for 40 min at 4 °C and 10,000 rpm. The supernatant phase was collected and filtrated through a membrane disc (47 mm diameter and 0.45 μm pore size). The proteins present in the fraction were precipitated with an initial concentration of (NH_4_)_2_SO_4_ at 65%. The remaining proteins in the fraction were precipitated at a salt saturation of 85%. Ammonium was added under constant agitation at 4 °C. Precipitated proteins were separated during the second period of centrifugation (10,000 rpm, 4 °C, 40 min). The obtained pellet was dissolved in a minimum volume of a sodium acetate buffer (10 mM, pH 5.0) and dialyzed overnight at 4 °C and constant agitation [12].

### 2.9. Purification of α-Amylase LB04 by Ion Exchange Chromatography

The dialyzed solution from the previous step was transferred to a column (20 × 400 mm) loaded with a DEAE-Sephadex matrix. The column was pre-equilibrated with a 20 mM acetate buffer, pH 6.0. Fractions were collected at a flow rate of 0.5 mL per minute against a gradient of NaCl from 0 to 500 mM. The obtained fractions were evaluated for protein content and enzyme activity.

### 2.10. Electrophoretic Analysis of Purified Enzyme and Zymography

To determine the molecular weight of the α-amylase produced by *B. licheniformis* strain LB04, an SDS-PAGE was performed, employing a 12% running gel and a 5% stacking gel [22]. Samples were mixed with 10 μL loading buffer and loaded onto the gel. Electrophoresis was performed using a BIO-RAD Mini Protean-Tetra Cell with a voltage of 20 mA during the run. The protein bands were detected by Coomassie Brilliant Blue R250. Zymography for detection of α-amylase activity was performed by following the methodology proposed by Upadhyay [23].

### 2.11. Characterization of Enzymatic Properties of Purified α-Amylase LB04

#### 2.11.1. Thermostability and Acidic Resistance on α-Amylase Activity

In order to determine the optimal pH for the obtained α-amylase, the enzyme-containing fraction was tested in a pH range of 3.0–8.0, employing a 0.1 M glycine-HCl buffer (pH 3.0), sodium acetate buffer 0.1 M (pH 4.0–6.0) and a Tris-HCl buffer (pH 8.0), in a solution of 1.0% starch as substrate. The samples were incubated for 60 min at 40 °C. The activity and optimal temperature of the purified enzyme was measured through the DNS method by ranging temperatures from 20 to 90 °C during the incubation period employing a sodium acetate buffer 0.1 M pH 4.0 and a starch 1.0% solution as substrate.

#### 2.11.2. Effects of Calcium Ions on the Enzymatic Activity of α-Amylase

The calcium ion-dependency for hydrolytic activity of the α-amylase was analyzed employing a sodium acetate buffer 0.1 M, pH 4.0. The metallic ions present in the medium were chelated adding EDTA until a final concentration of 1.0 mM. The reaction conditions were the same as in the thermostability and acidic resistance activity test. The enzyme’s total activity (100%) was determined by reducing sugar production using the DNS method.

### 2.12. Immobilization of α-Amylase LB04 on Agar-Agarose Beads

Agar-agarose beads were formed from a solution of 1.0% (*w*/*v*) agarose and 4.0% (*w*/*v*) agar in a 25 mM sodium acetate buffer solution. The pH in solution was adjusted to 5.5 and heated to 50 °C to solubilize the agar and the agarose. Once homogenized, a volume of 9.0 mL was mixed with 1.0 mL of protein solution at a fixed concentration of 0.5 mg of protein per milliliter. The mixture was poured into a sterile glass plate until it had completely solidified. Afterward, the gel was cut into beads of 5 × 5 mm and washed with sodium acetate buffer 25 mM. The buffer was collected for protein quantification [16].

### 2.13. Immobilized Enzyme Assay

The binding of α-amylase into the agar-agarose beads was analyzed through a DNS assay. Beads were suspended in 0.5 mL of 10 mM sodium acetate buffer and 0.5 mL of a 1.0% soluble starch solution. This enzyme binding reaction was performed at pH 3.0, 4.0, 5.0 and 6.0, for 30 min at 65 °C. These analytical parameters were designed to be within the range that resulted in the optimal α-amylase activity as shown in a previous test. Every 5 min, subsamples of 50 μL were collected, stopping the reaction by adding 50 µL of DNS solution. The subsamples were boiled for 5 min and cooled down at 4 °C for 10 min, then mixed with 500 μL of deionized water. Following the methodology established in the DNS assay for amylase activity determination [20], the concentration of the reducing sugars in the samples was analyzed through spectrophotometry at 540 nm, where 1 unit of α-amylase activity was defined as the amount of enzyme needed to produce 1 µmol of maltose per min from soluble starch at 65 °C.

## 3. Results

### 3.1. Identification and Starch-Degrading Activity of the Strain LB04

Of the 14 samples collected, 10 isolates showed the ability to grow in starch agar after 24 h of incubation at 45 °C. However, only 4 strains produced halo zones in the agar plates and were consequently selected to produce amylases to study their activity profile. A strain labeled LB04 was identified as Gram-positive, rod-shaped bacteria and exhibited growth and a production profile for an α-amylase that maintained amylolytic activity at low pH ranges and high temperatures. A summary of the biochemical characteristics observed for LB04 is shown in Table 1.

**Figure 1:**
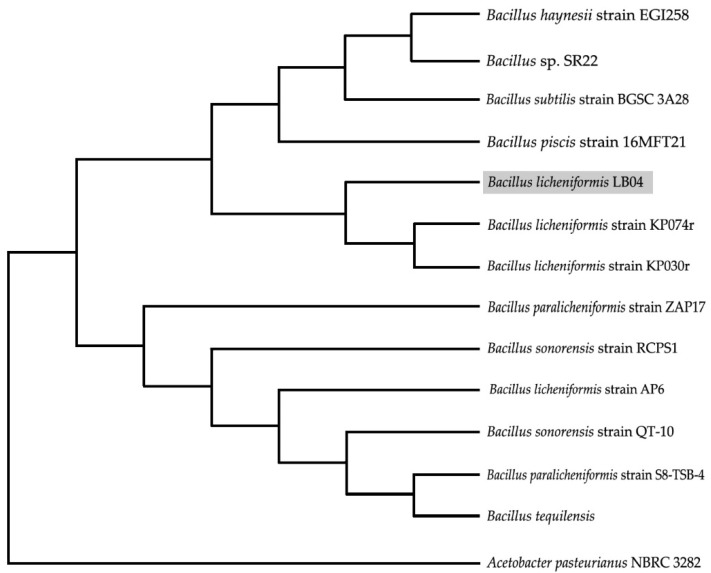
Molecular identification based on 16S rDNA sequence. The phylogenetic tree was constructed by a neighbor-joining algorithm based on the 16S rDNA gene sequences of *B. licheniformis* strain LB04 against related species of *Bacillus*.

A further analysis of the 16S rDNA gene segment amplification and using the bioinformatics tool, BLAST, indicated the strain LB04 to be *Bacillus licheniformis* with a 99.8% identity, as compared with the NCBI website database (Figure 1).

The *B. licheniformis* strain LB04 showed starch hydrolysis halos around the colonies while incubated at 60 °C, indicating thermostability due to the production of α-amylases, commonly associated with thermotolerant performing enzymes [24].

### 3.2. Growth Rate of Bacillus licheniformis Strain LB04

**Figure 2:**
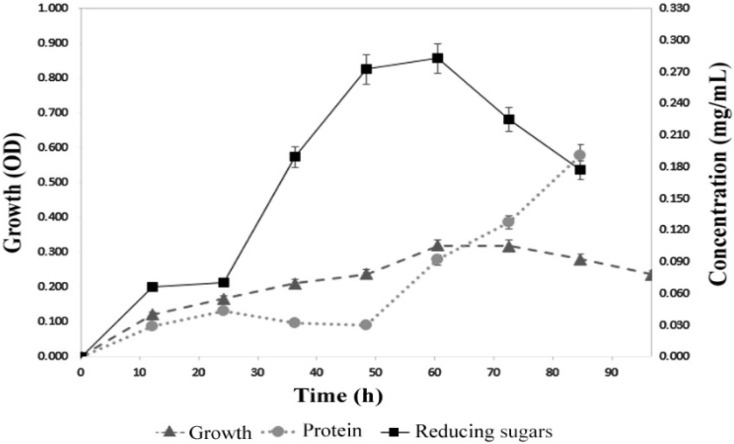
Growth chart of the *B. licheniformis* strain LB04. Conditions of growth: 45 °C, 150 rpm, in soluble starch medium.

The *B. licheniformis* strain LB04 growth was monitored for 90 h, taking samples for analysis every 12 h. The stationary phase lasted from 48 to 72 h, followed by a rapid decline in the growth rate. Similar time patterns were previously reported for different *B. licheniformis* strains [25]. As shown in Figure 2, the presence of reducing sugars was registered since the first 12 h, reaching the maximum point at 60 h, with 0.28 mg mL^−1^ ± 0.02 of free reducing sugars in the supernatant fraction.

### 3.3. Production of α-Amylase by B. licheniformis Strain LB04

**Figure 3:**
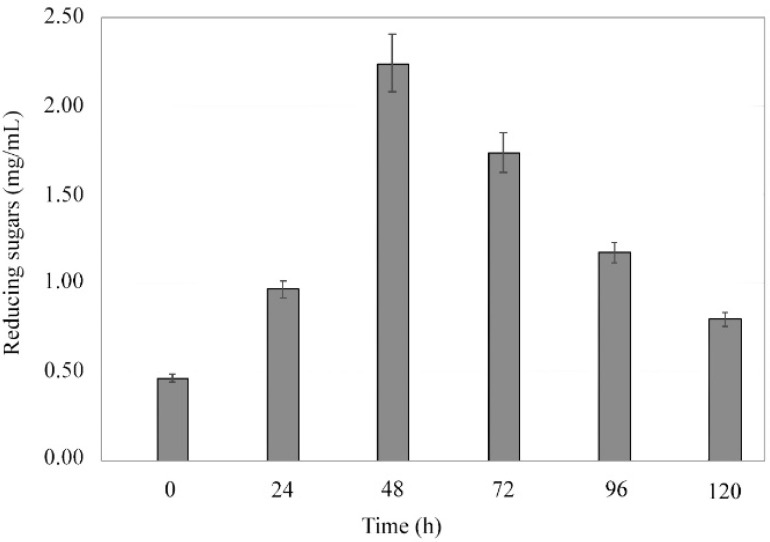
Release of reducing sugars during the fermentation process by *B. licheniformis* strain LB04. Maximum production levels of α-amylase reached at 48 h (2.35 mg mL^−1^ ± 0.15).

Reducing sugar production peaked at 48 h (2.35 mg mL^−1^ ± 0.15), equivalent to 134.8 U (Figure 3). *Bacillus licheniformis*, in accordance to similar species, was reported to reach maximum production levels of α-amylase by 48 h; after that, the presence of the reducing sugars in the medium declines due to accumulation of by-products and the depletion of nutrients [26,27].

### 3.4. Purification of α-Amylase

A recovery of 0.8 mg of purified α-amylase was obtained, as can be seen in the protein purification chart in Table 2.

**Figure 4:**
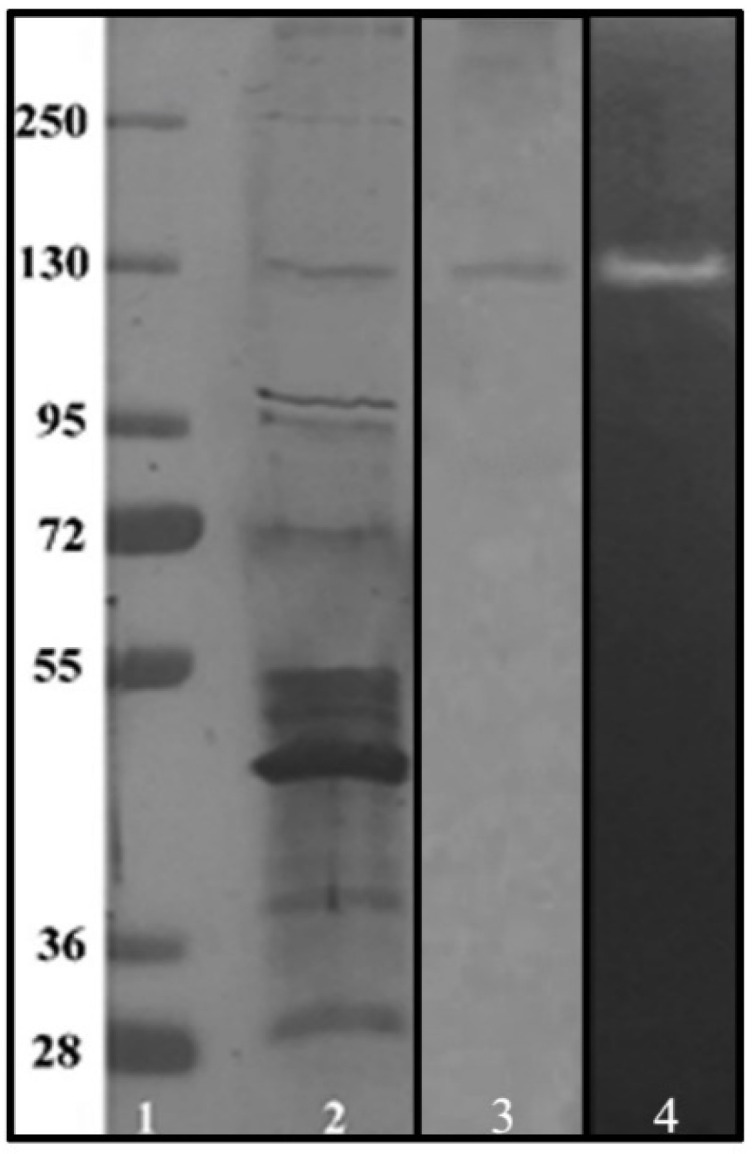
SDS-PAGE of the protein composition of different samples from *B. licheniformis* strain LB04. Lane 1—Molecular weight marker. Lane 2—Composition of the crude extract at 48 h of fermentation. Lane 3—Fraction 33, eluted at 250 mM NaCl. Lane 4—Zymogram of fraction 33 for detection of α-amylase activity.

The eluted fractions at 250 mM NaCl showed amylase activity. Analysis by SDS-PAGE and zymography confirmed the presence of a protein with a molecular weight of nearly 130 kDa and starch-degrading activity, as seen in Figure 4.

### 3.5. High Temperature and pH Ranges of Activity for the α-Amylase Produced by Strain LB04

**Figure 5:**
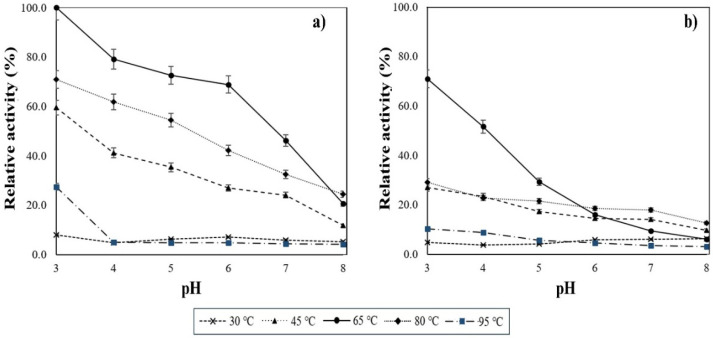
(**a**) Purified α-amylase under different temperatures and pH levels. (**b**) Purified α-amylase under different temperatures and pH levels with the addition of EDTA (1.0 mM) to chelate calcium ions. The maximum activity (100%) was equivalent to 1851.7 U mg^−1^ ± 1.3.

The purified enzymatic fraction was tested under a range of temperatures and pH levels. The influence of these parameters can be seen in Figure 5. From 30 to 95 °C, the peek activity was shown at 65 °C under acidic conditions (pH 3.0), with a specific activity of 1851.7 U mg^−1^ ± 1.3. By raising the pH level, a drop in enzymatic activity could be observed from pH 4.0 to 8.0. As the reaction was performed under alkaline conditions, the residual activity significantly decreased to 20.6% of the original value.

### 3.6. Influence of Calcium Ions on the Enzymatic Activity

The purified α-amylase enzyme was analyzed through a DNS assay and by adding EDTA as a chelating agent. The analysis was conducted as in previous conditions (data shown in Figure 5b). A distinctive decrease in the enzymatic activity for most of the temperature and pH ranges was shown after the addition of EDTA. It was noticed that the enzymatic activity at 65 °C and pH 3.0, despite being the point of highest enzymatic activity, dropped nearly 25% after the addition of EDTA when compared to chelating-free conditions. The same trend was present at all temperatures. Still, none of the activity points were completely inhibited by the chelation of the calcium ions.

### 3.7. Immobilization of α-Amylase on Agar-Agarose Beads

**Figure 6:**
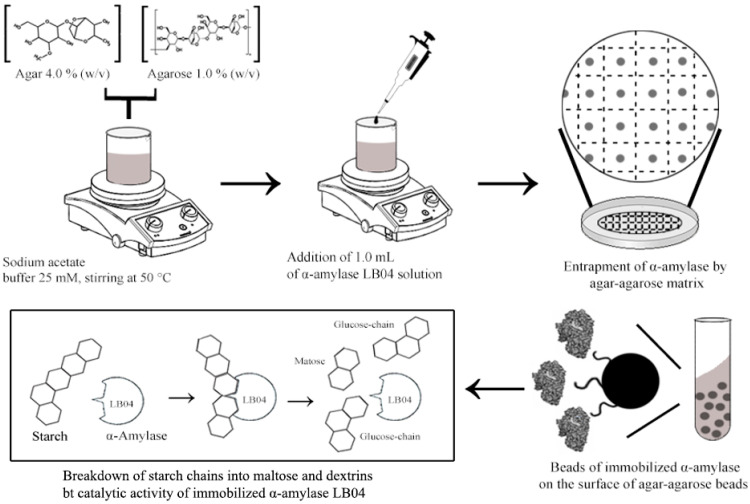
Representation of the process followed for entrapment of the α-amylase produced by *B. licheniformis* LB04, on the surface of the agar-agarose beads.

A simplified representation of the step-by-step process of the α-amylase immobilization in agar-agarose beads is shown in Figure 6. During this process the beads entrap the enzyme on its surface, allowing a better interaction with the substrate. However, from the initial 0.5 mg of α-amylase used for entrapment in the agar-agarose beads, only 0.36 ± 0.04 mg were immobilized after washing the beads with the 25 mM sodium acetate buffer, equivalent to an estimated of 0.031 mg of α-amylase per gram of beads. The protein loading efficiency was calculated to be 72.1%, with an entrapment yield of 19.9%. This might suggest further analysis of different polymers in order to achieve greater compatibility with the α-amylase produced by *B. licheniformis* strain LB04 [28].

### 3.8. Acidic Stability of Immobilized α-Amylase

**Figure 7:**
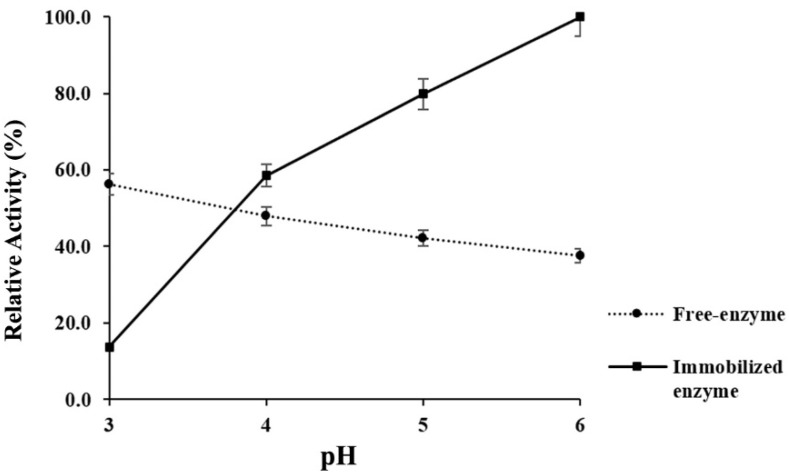
Response of the α-amylase during chelating conditions under a reaction temperature of 65 °C and different pH levels. Comparison between the enzymatic activity shown by the free form of the enzyme and its immobilized form. The maximum activity (100%) was equivalent to 1851.7 U mg^−1^ ± 1.3.

As we can see in Figure 7, immobilization of α-amylase in agar-agarose beads resulted in enhanced enzymatic activity at pH 6. The increase in activity shown by the immobilized enzyme was almost three times greater when compared to the free enzyme, even under the same conditions. On the other hand, the change in the structure of the enzyme due to the immobilization affected the acid-stability, considerably decreasing the enzymatic activity at pH 3.0. The free non-immobilized enzyme showed better amylolytic performance at lower pH values, with a gradual activity decrease as pH approached neutrality. After the immobilization process, the acid-stability was only 202.4 U mg^−1^, when compared to the original 834.1 U mg^−1^.

## 4. Discussion

During the growth study, the protein levels released to the supernatant fraction followed the growth curve at low concentrations from 12 to 48 h (less than 0.03 mg mL^−1^ ± 0.005) (Figure 2), indicating the production of α-amylase as a primary metabolite since its production was growth-related to the carbon source (soluble starch) [29]. Once increased, dextrins are released to the medium. In response, the bacteria shift from the starch as the main source to the use of reduced sugars. Afterward, a rapid depletion of glucose and maltose is observed. This pattern is related to the catabolite repression (CR) in *B. licheniform* is due to the accumulation of degradation products such as glucose. The expression of enzymes like α-amylase leads to the buildup of reduced sugars in the medium and the later repression of *B. licheniformis* promoters, preventing the amylase over-expression [30,31]. Once the 60-h threshold is passed, the CR is observed as the carbon source changes to glucose and enzymes non-related to starch degradation are produced.

It has been reported that commonly, the molecular mass for enzymes such as α-amylases ranges from 55 to 70 kDa [30]. However, some amylase enzymes produced by diverse *Bacillus* sp. strains have been reported to have molecular weights ranging from 130 to 210 kDa, which is associated with several factors, such as thermostable conformations of wild-type enzymes [32], pH acid-alkaline resistance [33,34] and the type of carbon-source (substrate) [35]. The heat and acidic resistance are one of the main characteristics observed in LB04 α-amylase, as the enzyme demonstrated higher enzyme activity levels at lower pH points and higher temperatures. The activity profile can be observed in Figure 5, where the lower the pH in the reaction the higher enzyme activity. The drop in activity is shown as the reaction becomes alkaline. This is suggested to be caused by changes in the enzyme conformation [36]. The alkaline adaptation of extracellular enzymes produced by microorganisms is rare since the intracellular medium has neutral pH. However, remodeling of amino acid pairs such as Arg-Asp in α-amylase was shown to increase alkaline performance, while Lys-Asp pairing was linked to non-alkaline resistance [37]. The latter configuration could be linked to the nature of the α-amylase produced by *B. licheniformis* strain LB04. Nonetheless, the structure of the enzyme must be provided for a deeper understanding of the thermo and acidic stability of the obtained α-amylase.

The study of the calcium ions’ influence over α-amylase activity, demonstrated how enzymatic activity is partially reduced by EDTA additions. These results agree with previous studies on the Ca^2+^-independent α-amylase produced by native *B. licheniformis* species [38,39], indicating a partial independence from calcium ions due to a continuous starch hydrolysis despite a lower efficiency [40,41]. As pH increased, the activity dropped; an effect that could be attributed to the enzyme’s structure configuration, more specifically due to irregularities in segments belonging to the loop regions that participate in the metal-binding zones 177–199 [41]. Amino acids in the domain B region were reported to play a fundamental role in thermostability [42]. The study of these regions is necessary for further conclusions; however, a similar performance profile was reported for neutrophilic and acidophilic endo-β-glucanases with a similar fold in their catalytic domain [43]. Nevertheless, the factors that establish the acidophilic nature of α-amylase are still unanswered, and further research is imperative to acquire this knowledge.

Through the results obtained it is clear that the immobilization process affected the activity profile of the α-amylase, since the acidic stability assay of immobilized amylase showed a discordance of activities. While the free enzyme showed a better acidic resistance, its immobilized form suffered large reductions of activity under the same conditions, and the activity increased as the reaction medium became alkaline. This profile is not exclusive to the α-amylase produced by *B. licheniformis* strain LB04, as previous research reported increased enzyme activity after immobilization by entrapment, with marked drops at points where originally there were higher values [44,45,46]. The acid-stability for immobilized α-amylases is attributed to structural preservation, limiting the expansion of the enzyme’s domains during the increase in temperatures and change in pH [47].

In order to avoid altering the active sites of α-amylase LB04, the selected immobilization method was entrapment. The entrapment technique is a physical method where the enzyme is confined within the polymer network of the matrix. For this study the matrix were beads made out of agar-agarose, a polymer with a strong gelling activity that shows no reaction with other biomolecules. However, this biopolymer is not suitable for studies at higher temperatures, since beads start to disintegrate beyond 75 °C. [48]. In order to study the enzymatic activity of the immobilized α-amylase at higher temperatures, a different carrier must be selected.

The activity of immobilized enzymes depends on factors such as the immobilization method and the nature of the matrix. Based on the present results, a different strategy must be approached, since although the agar-agarose beads improved the heat stability of the α-amylose LB04 at 65 °C, it was at higher pH levels as compared to the free form. This pattern was previously reported, where immobilization of amylases in a silica-based matrix during acid catalyzed sol-gel entrapment lowered the activity of the enzyme instead of enhancing it [49]. With similar results, agar gel entrapment improved the performance of different enzymes at neutral and alkaline conditions, but decreased the enzymatic activity of the free enzyme at lower pH levels [50]. While the entrapment technique is a straightforward method that preserves the enzyme conformation and the agar is a low-cost polymer with a non-toxic nature, in order to improve the acidic profile of the α-amylase produced by *B. licheniformis* LB04, different techniques and resources should be employed. A matrix like chitosan, which is an abundant, non-toxic and inert polysaccharide, is one of the most popular options for enzymatic immobilization. The amines and hydroxyl groups in this polymer allow crosslinking with the enzyme and different supports. Since chitosan beads previously improved the acidic hydrolysis of the α-amylase-by 50%, they might become a suitable strategy for further analysis of hydrolytic enhancement by immobilization [51].

To fully associate these observations as a peculiarity of the α-amylases produced by the *Bacillus* genus, due to the nature of α-amylases immobilization by entrapment or due to the agar-agarose support, further tests are necessary.

## 5. Conclusions

This study revealed the capacity of *B. licheniformis* strain LB04, a bacterium adapted to tolerate continuous environmental extremes in temperature and pH, to produce a versatile α-amylase capable of performing starch hydrolysis at temperatures above 65 °C and acidic conditions. The results suggest that the isolated bacteria and its α-amylase could be employable under conditions commonly met in industrial processes, especially in the refined syrups and bakery industries.

However, further studies on the enzyme activity are required to determine its potential in real manufacturing applications. While immobilization is a perfect tool for improving and recovering the enzyme, in this particular case, a different immobilization technique should be employed due to the loss of the amylolytic activity during acidic conditions and high temperatures. These characteristics are crucial for industrial processes where conditions are too harsh for most of the available commercial enzymes. While the improvement of a better-suited immobilization technique is important, new information related to the enzyme structure must be acquired to understand how the enzyme adapts to extreme conditions.

## Figures and Tables

**Table 1:** Identification profile of *Bacillus licheniformis* strain LB04.

Characterization of the LB04 Strain		
	Test	LB04
Colony character	Color	White
	Colony Shape	Irregular
	Edge	Undulate
	Elevation	Flat
Morphology	Gram character	+
	Shape	Rod
	Arrangement	Single
	Motility	−
Biochemical profile	Amylase	+
	Protease	+
	Lipase	+
	Catalase	+
	Oxidase	−
	Indole	−
	Gas production	−
	SH_2_ production	−
	Glucose	+
	Fructose	−
	Lactose	−
Environmental tolerance	Temp. tolerance range	20–90 °C
	pH tolerance range	2.0–10.0

Positive test results, labeled with “+” sign, negative results labeles with “−“.

**Table 2:** Protein purification steps for α-amylase produced by *B. licheniformis* strain LB04. The experiments were performed in triplicate.

Step	Total Activity (U)	Total Proteins (mg)	Specific Activity (U/mg)	Purification (Fold)	Yield (%)
Crude extract	14,450 ± 142.7	44.0	328.4 ± 6.5	1.0	100
Ammonium sulfate precipitation (65%)	6647 ± 98.2	10.0	664.7 ± 10.6	2.0	46
Ammonium sulfate precipitation (85%)	4018 ± 34.9	2.8	1435 ± 27.8	4.4	28
DEAE-Sepharose	3092 ± 12.1	0.8	3865 ± 47.2	11.8	21.4

## Data Availability

The authors confirm that the data supporting the findings of this study are available within the article.

## References

[1] Robertson G., Wong D., Lee C., Wagschal K., Smith M.R., Orts W.J. (2006). Native or raw starch digestion: A key step in energy efficient biorefining of grain. J. Agric. Food Chem..

[2] Peng H., Chen M., Yi L., Zhang X., Wang M., Xiao Y., Zhang N. (2015). Identification and characterization of a novel raw-starch degrading α-amylase (AmyASS) from the marine fish pathogen *Aeromonas salmonicida* ssp. salmonicid. J. Mol. Catal. B Enzym.

[3] Straksys A., Kochane T., Budriene S. (2016). Catalytic properties of maltogenic α-amylase from *Bacillus stearothermophilus* immobilized onto poly(urethane urea) microparticles. Food Chem..

[4] Saini R., Saini H., Dahiya A. (2017). Amylases: Characteristics and industrial applications. J. Pharmacogn. Phytochem..

[5] Simair A.A., Qureshi A.S., Khushk I., Ali C.H., Lashari S., Bhutto M.A., Mangrio G.S., Lu C. (2017). Production and Partial Characterization of α-Amylase Enzyme from *Bacillus* sp. BCC 01-50 and Potential Applications. BioMed Res. Int..

[6] Alpha-Amylase Baking Enzyme Market to Reach USD 347.7 Million by 2026|Reports and Data. https://www.globenewswire.com/newsrelease/2019/04/25/1809974/0/en/Alpha-Amylase-Baking-Enzyme-Market-To-Reach-USD-347-7-Million-By-2026-Reports-And-Data.html.

[7] Barrera G., Tadini C., León A., Ribotta P. (2016). Use of alpha-amylase and amyloglucosidase combinations to minimize the bread quality problems caused by high levels of damaged starch. J. Food Sci. Technol..

[8] Purhagen J., Sjöö M., Eliasson A. (2011). Starch affecting anti-staling agents and their function in freestanding and pan-baked bread. Food Hydrocoll..

[9] Sen S., Jana A., Bandyopadhyay P., Das-Mohapatra P., Raut S. (2016). Thermostable amylase production from hot spring isolate *Exiguobacterium* sp.: A promising agent for natural detergents. Sustain. Chem. Pharm..

[10] Sundarram A., Murthy T. (2014). α-Amylase Production and Applications: A Review. J. Appl. Environ. Microbiol..

[11] Bouacem K., Laribi-Habchi H., Mechri S., Hacene H., Jaouadi B., Bouanane-Darenfed A. (2018). Biochemical characterization of a novel thermostable chitinase from *Hydrogenophilus hirschii* strain KB-DZ44. Int. J. Biol. Macromol..

[12] Allala F., Bouacem K., Boucherba N., Azzouz Z., Mechri S., Sahnoun M., Benallaoua S., Hacene H., Jaouadi B., Bouanane-Darenfed A. (2019). Purification, biochemical, and molecular characterization of a novel extracellular thermostable and alkaline α-amylase from *Tepidimonas fonticaldi* strain HB23. Int. J. Biol. Macromol..

[13] Basso A., Serban S. (2019). Industrial applications of immobilized enzymes—A review. Mol. Catal..

[14] Mohamed S., Khan A., Al-Bar A., El-Shishtawy R. (2014). Immobilization of *Trichoderma harzianum* α-amylase on treated wool: Optimization and characterization. Molecules.

[15] Mohamed S., Al-Harbi M., Almulaiky Y., Ibrahim I., Salah H., El-Badry M., Abdel-Aty A., Fahmy A., El-Shishtawy R. (2018). Immobilization of *Trichoderma harzianum* α-amylase on PPyAgNp/Fe_3_O_4_-nanocomposite: Chemical and physical properties. Art. Cells Nanomed. Biotechnol..

[16] Prakash O., Jaiswal N. (2011). Immobilization of a thermostable α-amylase on agarose and agar matrices and its application in starch stain removal. World Appl. Sci. J..

[17] Blanco de la Cruz L. (2016). Glycerol Bioconversion by Thermotolerant Microorganism Isolated from Northeast Mexico. Master’s Thesis.

[18] Minor V. (1928). Ein Neues Verfahren zu der Klinischen Untersuchung der Schweißabsonderung. Dtsch. Z. Nervenheilkd.

[19] Deljou A., Arezi I. (2016). Production of thermostable extracellular α-amylase by a moderate thermophilic *Bacillus licheniformis*-AZ2 isolated from Qinarje Hot spring (Ardebil prov. of Iran). Period. Biol..

[20] Miller G. (1959). Use of Dinitrosalicylic Acid Reagent for Determination of Reducing Sugar. Anal. Chem..

[21] Bradford M. (1976). A rapid and sensitive method for the quantitation of microgram quantities of protein utilizing the principle of protein-dye binding. Anal. Biochem..

[22] Laemmli U. (1970). Cleavage of structural proteins during the assembly of the head of bacteriophage T4. Nature.

[23] Upadhyay M., Sharma R., Pandey A., Rajak R. (2005). An improved zymographic method for detection of amylolytic enzymes of fungi on polyacrylamide gels. Mycologist.

[24] Gamal R., Abou-Taleb K., Abd-Elhalem B. (2017). Isolation, Identification and Production of Amylases from Thermophilic Spore Forming Bacilli Using Starch Raw Materials Under Submerged Culture. AASCIT J. Biosci..

[25] El-Sheshtawy H., Aiad I., Osman M., Kobisy A., El-Sheshtawy H., Abo-Elnasr A. (2015). Production of biosurfactant from *Bacillus licheniformis* for microbial enhanced oil recovery and inhibition the growth of sulfate reducing bacteria. Egypt. J. Pet..

[26] Hiteshi K., Didwal G., Gupta R. (2016). Production optimization of α-amylase from *Bacillus licheniformis*. J. Adv. Res. Biol. Pharm. Res..

[27] Alariya S., Sethi S., Gupta S., Lal-Gupta B. (2013). Amylase activity of a starch degrading bacteria isolated from soil. Arch. Appl. Sci. Res..

[28] Chaudhary M., Rana N., Vaidya D., Ghabru A., Rana K., Dipta B. (2019). Immobilization of Amylase by Entrapment Method in Different Natural Matrix. Int. J. Curr. Microbiol. Appl. Sci..

[29] Esfahanibolandbalaie Z., Rostami K., Mirdamadi S. (2008). Some studies of α-amylase production using *Aspergillus oryzae*. Pak. J. Biol. Sci..

[30] Nathan S., Nair M. (2013). Engineering a repression-free catabolite-enhanced expression system for a thermophilic alpha-amylase from *Bacillus licheniformis*. J. Biotechnol..

[31] Russell J., Baldwin R. (1978). Substrate preferences in rumen bacteria: Evidence of catabolite regulatory mechanisms. Appl. Environ. Microbiol..

[32] Maktouf S., Kamoun A., Moulis C., Remaud-Simeon M., Châabouni S. (2013). A new raw-starch-digesting α-amylase: Production under solid-state fermentation on crude millet and biochemical characterization. J. Microbiol. Biotechnol..

[33] Burgess-Cassler A., Imam S., Gould J. (1991). High-molecular-weight amylase activities from bacteria degrading starch-plastic films. Appl. Environ. Microbiol..

[34] Abou-Dobara M., El-Sayed A., El-Fallal A., Omar M. (2011). Production and partial characterization of high molecular weight extracellular α-amylase from *Thermoactinomyces vulgaris* isolated from Egyptian soil. Pol. J. Microbiol..

[35] Timilsina P., Pandey G., Shrestha A., Ojha M., Karki T. (2020). Purification and characterization of a noble thermostable algal starch liquefying α-amylase from *Aeribacillus pallidus* BTPS-2 isolated from geothermal spring of Nepal. Biotechnol. Rep..

[36] Behal A., Singh J., Sharma M., Puri P., Batra N. (2006). Characterization of alkaline α-amylase from *Bacillus* sp. AB 04. Int. J. Agric. Biol..

[37] Pinto E., Dorn M., Feltes B. (2020). The tale of a versatile enzyme: α-amylase evolution, structure, and potential biotechnological applications for the bioremediation of n-alkanes. Chemosphere.

[38] Matpan-Bekler F., Acer Ö., Güven K. (2015). Co-Production of Thermostable, Calcium-Independent α-Amylase and Alkali-Metallo Protease from Newly Isolated *Bacillus licheniformis* Dv3. Innov. Rom. Food Biotechnol..

[39] Du R., Qiaozhi S., Qiaoge Z., Zhao F., Kim R., Zhou Z., Han Y. (2018). Purification and characterization of novel thermostable and Ca-independent α-amylase produced by *Bacillus amyloliquefaciens* BH072. Int. J. Biol. Macromol..

[40] Hmidet N., Bayoudh A., Berrin J., Kanoun S., Juge N., Nasri M. (2008). Purification and biochemical characterization of a novel α-amylase from *Bacillus licheniformis* NH1. Cloning, nucleotide sequence and expression of amyN gene in *Escherichia coli*. Process Biochem..

[41] Wu H., Tian X., Dong Z., Zhang Y., Huang L., Liu X., Jin P., Wang Z. (2018). Engineering of *Bacillus amyloliquefaciens* α-Amylase with Improved Calcium Independence and Catalytic Efficiency by Error-Prone PCR. Starch-Stärke.

[42] Li Z., Duan X., Chen S., Wu J. (2017). Improving the reversibility of thermal denaturation and catalytic efficiency of *Bacillus licheniformis* α-amylase through stabilizing a long loop in domain B. PLoS ONE.

[43] Huang Y., Krauss G., Cottaz S., Driguez H., Lipps G. (2005). A highly acid-stable and thermostable endo-β-glucanase from the thermoacidophilic archaeon *Sulfolobus solfataricus*. Biochem. J..

[44] Talekar S., Chavare S. (2012). Optimization of immobilization of α-amylase in alginate gel and its comparative biochemical studies with free α-amylase. Recent Res. Sci. Technol..

[45] Sharma M., Sharma V., Majumdar D. (2014). Entrapment of α-Amylase in Agar Beads for Biocatalysis of Macromolecular Substrate. Int. Sch. Res. Not..

[46] Tavano O., Fernandez-Lafuente R., Goulart A., Monti R. (2013). Optimization of the immobilization of sweet potato amylase using glutaraldehyde-agarose support. Characterization of the immobilized enzyme. Process Biochem..

[47] Liu Y., Huang L., Jia L., Gui S., Fu Y., Zheng D., Guo W., Lu F. (2017). Improvement of the acid stability of *B. licheniformis* alpha amylase by site-directed mutagenesis. Process Biochem..

[48] Kalita T., Sangma S., Bez G., Ambasht P. (2020). Immobilization of Acid Phosphatase in Agar-agar and Gelatin: Comparative Characterization. J. Sci. Res..

[49] Mesbah N., Wiegel J. (2018). Improvement of Activity and Thermostability of Agar-Entrapped, Thermophilic, Haloalkaliphilic Amylase AmyD8. Catal. Lett..

[50] Fernandez-Caresani J., Dallegrave A., dos Santos J. (2020). Amylases immobilization by sol–gel entrapment: Application for starch hydrolysis. J. Sol-Gel Sci. Technol..

[51] Mardani T., Khibani M., Mokarram R., Hamishehkar H. (2018). Immobilization of α-amylase on chitosan-montmorillonite nanocomposite beads. Int. J. Biol. Macromol..

